# Multiple intelligence profiles of physical education teacher candidates

**DOI:** 10.3389/fpsyg.2025.1459051

**Published:** 2025-06-26

**Authors:** Çiğdem Karagülmez Sağlam, Erman Doğan

**Affiliations:** ^1^Department of Physical Education and Sports Teaching, Faculty of Sports Sciences, Girne American University, Girne, Cyprus; ^2^Department of Sports Management, Faculty of Sports Sciences, Girne American University, Girne, Cyprus

**Keywords:** teacher training, multiple intelligences, physical education, gender differences, school background, student teachers, sport type

## Abstract

This study investigates the multiple intelligence profiles of prospective physical education teachers and examines how these profiles vary by gender, high school type, and sport participation. A total of 102 fourth-year students (Mage = 21.45, SD = 1.88) enrolled in Physical Education and Sports Teaching Departments at universities in Kyrenia and Nicosia (Northern Cyprus) participated in the study. Data were collected via a validated self-report inventory based on Gardner’s theory of multiple intelligences. The findings revealed that verbal–linguistic intelligence had the highest average score, while bodily-kinesthetic intelligence ranked lowest. Although overall intelligence profiles were relatively balanced, significant differences were found in certain intelligence domains based on gender and school type. For example, males scored significantly higher in visual–spatial, bodily-kinesthetic, and naturalistic intelligences, while students from religious vocational high schools scored markedly higher in existential intelligence. These results highlight the relevance of tailoring teacher training programs to the dominant intelligence strengths of teacher candidates.

## Introduction

Teaching is a profession that seeks effective solutions to educational challenges and requires specialized pedagogical competencies. The global demand for highly qualified teachers particularly in developing countries remains a critical concern (Wallet, 2015). For education to be effective, it must consider learners’ individual characteristics and developmental needs.

Historically, education was shaped by teacher-centred models that often overlooked individual differences among students. However, modern pedagogical paradigms have shifted toward student-centred approaches that acknowledge cognitive diversity, including intelligence, as a central factor in learning (Baidi, 2022; Moncada and Mire, 2017; Serrat, 2017).

Among the most widely discussed frameworks addressing individual cognitive diversity is Howard Gardner’s Theory of Multiple Intelligences (MI). Gardner (1983, 1993, 1999, 2006) proposed that intelligence is not a single general ability, but rather a combination of relatively independent domains, each grounded in distinct biological bases and developmental pathways. These domains include verbal–linguistic, logical-mathematical, bodily-kinesthetic, interpersonal, intrapersonal, musical, visual–spatial, naturalist, and existential intelligences (McCoog, 2007; Al Jaddou, 2018). Gardner defined intelligence as the capacity to solve problems or create products that are valued within one or more cultural contexts.

The MI theory has been influential in promoting inclusive education and differentiated instruction. It has expanded the concept of intelligence beyond conventional psychometric definitions and encouraged educators to adopt a more holistic view of student potential (Kornhaber, 2022; Armstrong, 2006). This approach is particularly relevant to physical education (PE), where bodily-kinesthetic, interpersonal, and verbal intelligences play a prominent role in effective teaching and student engagement (Shuadi et al., 2020; Düzgün and Akkoç, 2021).

Despite its widespread adoption in educational discourse, MI theory remains controversial in terms of its empirical grounding, especially within higher education and teacher training contexts (Cerruti, 2013; Shearer, 2018; Lei et al., 2021). Critics argue that the theory lacks clear mechanisms for how each intelligence functions or interacts and does not offer specific instructional strategies tailored to individual intelligence types (Waterhouse, 2023; Haier and Jung, 2008). Gardner himself has noted that the MI framework should be understood as a conceptual guide rather than a prescriptive model, describing it as a “Rorschach test” for educators (Gardner, 1983, 1999).

Nonetheless, many educators regard MI theory as a valuable tool for fostering inclusive pedagogy and reflective teaching. Empirical research suggests that applying MI in classroom settings can enhance student motivation, engagement, and academic performance especially when integrated thoughtfully into instructional design (Chen et al., 2009; Kornhaber, 2019; Sherman et al., 2023). In teacher education programs, MI can also function as a reflective tool that helps pre-service teachers better understand their own teaching styles and the cognitive needs of diverse learners (Rousseau, 2021; Attwood, 2022).

This is especially critical in physical education, where teachers frequently draw on multiple intelligence types, particularly bodily-kinesthetic, interpersonal, and verbal–linguistic intelligences—to facilitate communication, movement, collaboration, and emotional regulation (Del Pino Medina et al., 2009; Al-Adawi et al., 2023). A growing body of research supports the integration of MI-based assessments in teacher training to help identify dominant intelligence domains and enhance pedagogical planning (Shore, 2004; Gul and Rafique, 2017). This approach may also increase teacher candidates’ sense of self-efficacy and improve instructional adaptability across diverse classroom contexts.

While this research offers meaningful insights, it also carries several limitations. First, the sample is limited to prospective physical education teachers, which restricts the generalisability of the findings to other subject areas. Second, the study relies on self-report instruments to assess intelligence, which may be influenced by bias or inaccuracies in self-perception. Third, other potentially influential factors such as socioeconomic background, personality traits, or teaching experience were not considered in the analysis. Additionally, the cross-sectional nature of the study captures data at a single point in time, limiting insights into the developmental trajectory of intelligence profiles. Finally, although grounded in MI theory, the study does not assess the direct impact of MI based instructional methods on learning outcomes, which would be a valuable area for future research.

Despite these limitations, this study aims to contribute to the existing literature by identifying the dominant intelligence types among physical education teacher candidates and examining how these profiles vary according to gender, type of high school, and sport participation. Based on the literature, the following hypotheses are proposed:

*H1*: The dominant intelligence domains among pre-service physical education teachers are bodily-kinesthetic, interpersonal, and verbal-linguistic intelligences.

*H2*: There are significant differences in intelligence profiles based on gender.

*H3*: The type of high school from which students graduated significantly affects their intelligence profiles.

*H4*: Type of sport participation (individual, team, or both) is associated with differences in intelligence domains.

The findings are expected to support more tailored and effective teacher education strategies by aligning training practices with the cognitive strengths and instructional needs of future educators.

## Materials and methods

### Research design

A quantitative research design was employed, specifically utilizing a general survey model to collect data. This model is commonly used to gather information from an entire population or a representative sample with the goal of making generalizable inferences (Kabadayı, 2009; Creswell, 2014).

A cross-sectional survey design was adopted for this study. The reporting of the research followed the STROBE (Strengthening the Reporting of Observational Studies in Epidemiology) guidelines for cross-sectional studies, ensuring methodological rigor and transparency (Von Elm et al., 2008; Sánchez-Martín et al., 2024). In accordance with the STROBE checklist, comprehensive details were provided concerning the sampling strategy, data collection procedures, variable definitions, and statistical analyses.

This research design was deemed appropriate for assessing the current multiple intelligence profiles of the participants and exploring their associations with selected demographic factors. As the study did not aim to establish causal relationships, the results were interpreted descriptively, focusing on identifying trends and patterns rather than inferring cause-and-effect outcomes.

### Participants

This study was conducted with senior students enrolled in the Physical Education and Sports Teaching departments at universities in the Turkish Republic of Northern Cyprus (TRNC). Since only five universities in the TRNC offer this program, the population size and the sample (*n* = 102) were limited, and thus the findings are not generalizable to a broader population.

Participants were selected using a convenience sampling method, mainly due to restrictions during the pandemic period, which necessitated the inclusion of students who were easily accessible to the researchers. Only the responses of students who completed the survey in full were included in the analysis.

The data collection process was coordinated with department heads and student advisors. The online survey was administered via a Google Forms link shared through official departmental WhatsApp groups managed by student advisors. Weekly reminders were sent to encourage participation and ensure timely completion.

Participants completed the survey voluntarily in an uncontrolled, unsupervised environment, typically from their personal devices such as smartphones or computers. While no direct supervision was possible due to the remote nature of data collection, participants were informed about the importance of providing honest and thoughtful responses. The estimated time to complete the survey was approximately 15–20 min. It was assumed that participants responded sincerely and that their responses reflected the characteristics of the target group.

### Data collection tools

During the data collection process, two instruments were employed in the study: “Personal Information Form” measuring general demographic information and the “Multiple Intelligence Scale.”

#### Personal information form

Developed by the researcher, this 15-item form collects demographic data including participants’ gender, type of high school attended, preferred sport branch (individual/team/both). The demographic variables were used to examine their possible influence on multiple intelligence domains.

#### Multiple intelligences inventory

To assess participants’ multiple intelligence levels, the study used the Multiple Intelligences Inventory developed by McClellan and Conti (2008) and adapted into Turkish by Babacan and Dilci (2012). The inventory is grounded in Gardner’s Theory of Multiple Intelligences and includes 27 items distributed across nine intelligence domains. The total score for each domain ranges from 3 to 27. Higher scores indicate a stronger inclination toward that specific intelligence domain. Each intelligence domain is represented by three items:

Bodily/Kinesthetic Intelligence: Items 1, 10, 19.
Existential Intelligence: Items 2, 11, 20.
Interpersonal Intelligence: Items 3, 12, 21.
Intrapersonal Intelligence: Items 4, 13, 22.
Logical-Mathematical Intelligence: Items 5, 14, 23.
Musical Intelligence: Items 6, 15, 24.
Naturalistic Intelligence: Items 7, 16, 25.
Verbal/Linguistic Intelligence: Items 8, 17, 26.
Visual–Spatial Intelligence: Items 9, 18, 27.

### Validity and reliability

The Turkish version of the inventory was validated through expert opinion for content validity, while construct validity was tested via factor analysis in the adaptation study conducted by Babacan and Dilci (2012). The adapted version preserved the structure of the original scale.

A 90-item draft scale was meticulously prepared in accordance with the recommendations set out by Howard Gardner on this particular subject. The students emphasized that some structures in the scale were complex in terms of readability, appearance and language used, and that the scale should not be used as a rating scale. They also suggested reducing the number of items, since they marked almost every item in the high range while answering. Following the consultation with students, it was agreed that the number of items would be reduced to 45. A correlation analysis was conducted in order to ascertain the contribution of each item to the total score. The scale was composed of 45 items, with the selection of the five items exhibiting the highest correlation for each intelligence area. A preliminary application of the 45-item version of the scale was conducted with 149 students enrolled in a special education programme. The mean total item score was 57.7%, with 26.7% of respondents achieving a score of 0.6 or above. The data presented herein were collected from a total of 787 students, who were selected via a random sampling method. In order to test the construct validity of the scale, 87 students were included in the study group in addition to the 787 students, and the responses of a total of 874 participants were processed. Upon examination of the exploratory factor analysis results, it was determined that the scale was distributed across 16 factors, with each factor exhibiting an Eigenvalue greater than 1. The first factor, which exhibited the highest magnitude of influence, accounted for 7.61% of the total variance, while the 16th factor contributed 2.22%. Utilizing the Multiple Intelligence Theory as a criterion, eight additional factor analysis processes were conducted, with the factor numbers ranging from 2 to 9 to generate nine factors. Consequently, the number of items was reduced to 27 in order to create 9 factors based on the Multiple Intelligence Theory. In the final version of the scale, 27 items were determined, consisting of three items with high loading values representing each intelligence area. Furthermore, it was determined that the correlation between the item total score was sufficiently high. The correlation values were as follows: one item was found to be above 0.80; 12 items were found to be between 0.70 and 0.79.9; 9 items were found to be between 0.60 and 0.69.9; and finally, 5 items were found to be between 0.50 and 0.59.9. The reliability of the multiple intelligence scale was obtained through the utilization of the test–retest method. The scale was administered to the students of the Faculty of Education at two-week intervals. Following a thorough analysis, it was determined that four of the nine intelligence areas demonstrated a score of 0.7 or above. The remaining four exhibited a slightly lower level of performance, with one area registering a score of 0.5. The scale was translated from English to Turkish by five experts in the field of foreign languages. Following the collection of opinions from five experts in the relevant field and two experts in Turkish, the Turkish version of the scale was created. Subsequent to this stage, the document was translated from Turkish to English by four experts in the field of foreign languages. Following a thorough examination, it was determined that there was no semantic discrepancy between the original version of the scale and the translation of the created Turkish form. In order to ascertain the linguistic validity of the scale, the original English version and the adapted Turkish version were administered to 130 undergraduate students studying in the Department of English Language and Literature. Furthermore, it was ascertained that the correlation between the item total score was sufficient. In this study, the overall internal consistency of the inventory was found to be Cronbach’s alpha = 0.95, indicating excellent reliability. Subscale alpha values were also above 0.70, demonstrating acceptable internal consistency across intelligence domains (Table 1).

**Table 1:** Reliability and validity indicators of the multiple intelligences inventory.

Type of analysis	Results
Kaiser-Meyer-Olkin (KMO) Measure	0.95
Bartlett’s Test of Sphericity	*p* < 0.05 (significant)
Cronbach’s Alpha (Overall Scale)	0.95 (high internal consistency)
Cronbach’s Alpha for Subscales	Ranges from 0.73 to 0.86 (acceptable levels)

### Data analysis procedures

Data were analyzed using SPSS 25.0, with statistical significance set at *p* < 0.05. Descriptive statistics were used to summarize demographic variables (Table 2).

**Table 2:** Cronbach’s alpha coefficients for each intelligence subscale.

Intelligence domain	Cronbach’s alpha
Bodily-Kinesthetic	0.85
Existential	0.85
Interpersonal	0.78
Intrapersonal	0.84
Logical-Mathematical	0.75
Musical	0.74
Naturalistic	0.73
Verbal–Linguistic	0.84
Visual–Spatial	0.86

For group comparisons:

- Independent samples t-tests were conducted to examine gender-based differences.
- One-way ANOVA was used to analyze differences in multiple intelligence domains across school types and sport branches.
- Where significant differences were found, Tukey’s post-hoc tests were applied to identify specific group differences.

While these analyses provide useful information, multivariate analyses (e.g., MANOVA, regression) will be addressed in other studies that will address interaction effects among variables.

### Ethical considerations

This study was approved by the Ethics Committee of the Faculty of Education at Girne American University (Approval No: 05/20–70, dated 25.09.2020). In addition, permission to administer the survey was granted by the Ministry of National Education of the Turkish Republic of Northern Cyprus (Approval No: MEB.0.00-006[006]-21/E.2282). All participants provided informed consent, and data were collected and processed anonymously in accordance with ethical research principles.

## Results

### Findings on demographic characteristics

Table 3 presents the gender distribution, type of high school attended, and preferred sport type of the participants. As illustrated, 26% of the physical education teacher candidates were female, while 74% were male. In terms of educational background, 31 participants (30.3%) graduated from general high schools, 23 (22.6%) from Anatolian or science high schools, 32 (31.3%) from vocational high schools, 2 (2%) from sports high schools, 5 (5%) from Imam Hatip (religious vocational) high schools, and 9 (8.8%) were university graduates. Regarding sports preference, 45 participants (44.1%) were primarily involved in individual sports, 35 (34.4%) in team sports, and 22 (21.6%) participated in both individual and team sports (Table 4).

**Table 3:** Data related to participants’ demographic characteristics.

		*N*
Gender	Male	26
	Female	76
School type	General High School	31
	Anatolian/Science High School	23
	Vocational High School	32
	Sports High School	2
	Religious Ed. High School	5
	College	9
Sports type	Individual	45
	Team	35
	Both	22

**Table 4:** Average and standard deviations of intelligence.

Intelligence	Mean	SD
Verbal	11.6569	5.56598
Mathematical/Logical	7.4608	5.58937
Visual	9.0784	4.95861
Musical/Rhythmic	9.6471	5.32256
Naturalistic	7.8431	5.655039
Interpersonal	7.8529	5.34622
Intrapersonal	7.4314	6.06811
Bodily/Kinesthetic	6.3333	5.66793
Existential	7.2255	5.53639

### Findings on average intelligence

An analysis of the average intelligence scores among physical education teacher candidates revealed that verbal/linguistic intelligence (11.6569) was the most dominant, while bodily/kinesthetic intelligence (6.3333) scores were notably lower. This result may reflect a curriculum orientation that places greater emphasis on theoretical instruction, verbal communication, and reflective practices rather than on the development of motor or physical skills. Additionally, these findings may be influenced by environmental factors, such as the COVID-19 pandemic, which restricted opportunities for physical activity and could have negatively impacted the development of kinesthetic intelligence. Overall, the candidates performed at comparable levels in the remaining intelligence areas.

**Figure 1:**
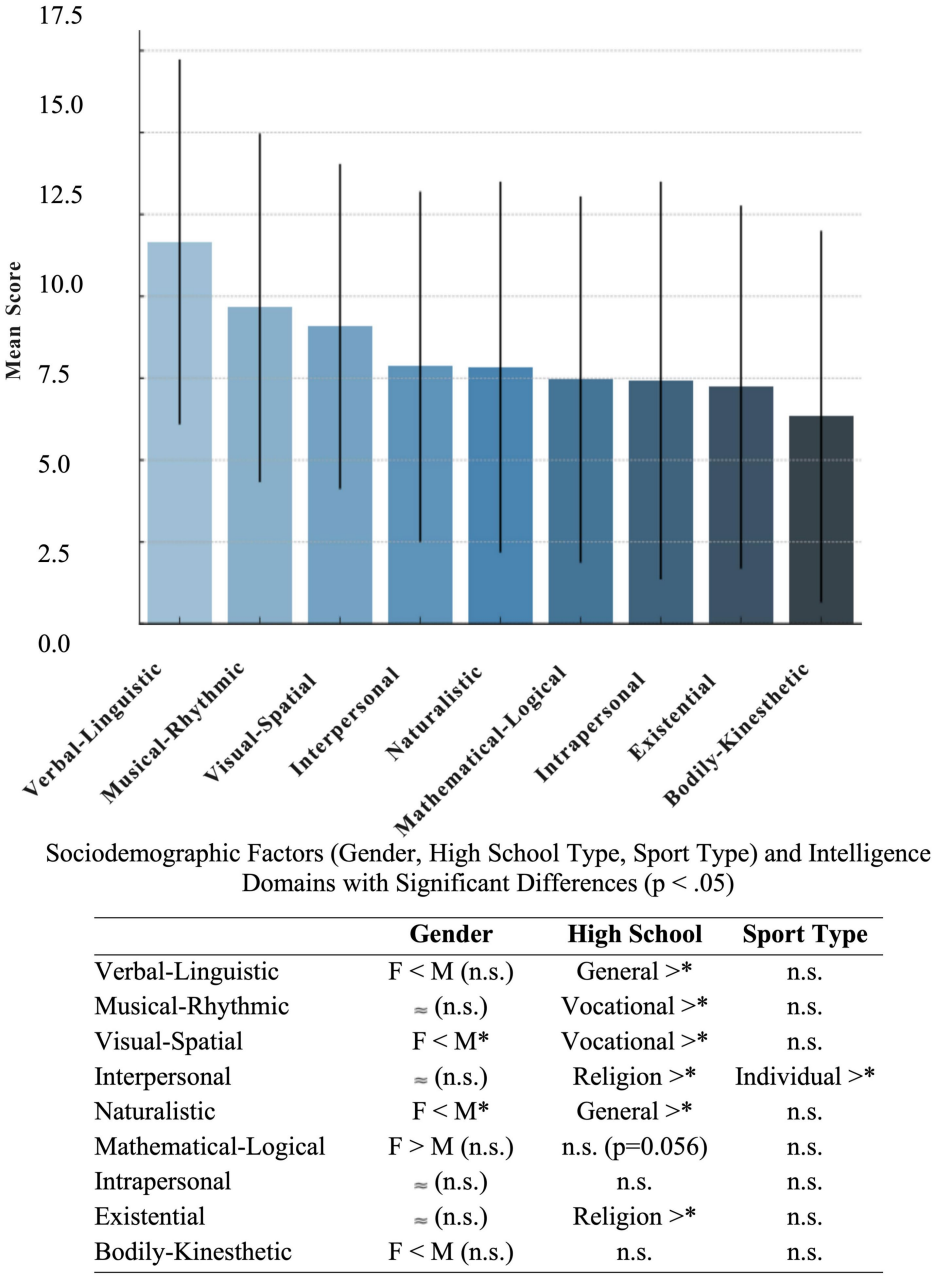
Mean scores of intelligence domains and their significant sociodemographic association.

These findings are further clarified in the figure below, which summarizes the average performance across intelligence domains along with sociodemographic factors that showed statistically significant differences (Figure 1).

Table 5 indicates that male physical education teacher candidates scored higher on kinesthetic/bodily, existential, naturalistic, verbal, and visual–spatial intelligence domains compared to their female counterparts. However, the differences were statistically significant only in the kinesthetic (*p* < 0.027), visual–spatial (*p* < 0.010), and naturalistic (*p* < 0.027) intelligence areas, suggesting a potential gender-related advantage in these domains (Table 6).

**Table 5:** Average of multiple intelligence areas by gender.

	Gender	*N*	Mean	SD Deviation	*T*	Sig. (2-tailed)
Bodily/Kinesthetic	Female	26	4.85	2.603	−1.561	0.027*
	Male	76	6.84	6.323		
Naturalistic	Female	26	5.77	3.993	−2.251	0.027*
	Male	76	8.55	5.846		
Visual	Female	26	6.92	3.877	−2.643	0.10*
	Male	76	9.82	5.093		

*Statistically significant difference *p* < 0.05.

**Table 6:** Average of multiple intelligence areas by graduated high school.

	High school	*N*	Mean	SD Deviation	*F*	*p*
Existential	General	31	6.87	5.336	*4.748*	0.001*
	Anatolian/Science	23	6.74	5.895		
	Vocational	32	6.19	5.375		
	Sport	2	4.00	1.414		
	Religion	5	17.60	8.829		
	College	9	8.33	6.728		
	College	9	8.22	5.505		
Intrapersonal	General	31	7.42	5.446	*2.882*	0.018*
	Anatolian/Science	23	6.43	6.013		
	Vocational	32	6.16	3.141		
	Sport	2	4.50	2.121		
	Religion	5	14.80	10.293		
	College	9	11.11	8.139		
Mathematical/ Logical	General	31	7.29	5.528	*2.378*	0.044*
	Anatolian/Science	23	7.13	5.457		
	Vocational	32	7.03	4.727		
	Sport	2	4.50	0.707		
	Religion	5	15.40	10.468		
	College	9	6.67	6.594		
Naturalistic	General	31	7.23	4.763	*3.047*	0.013*
	Anatolian/Science	23	8.26	6.618		
	Vocational	32	7.38	5.105		
	Sport	2	4.50	0.707		
	Religion	5	16.40	6.351		
	College	9	6.56	5.093		
Visual	General	31	9.19	4.752	*2.443*	0.039*
	Anatolian/Science	23	8.57	5.320		
	Vocational	32	8.59	4.543		
	Sport	2	8.00	5.657		
	Religion	5	16.20	7.471		
	College	9	8.00	4.176		

*Statistically significant difference *p* < 0.05.

The study also revealed statistically significant differences in intelligence levels across various domains based on the type of high school from which students graduated. For instance, a significant difference (*p* < 0.001) was observed in existential intelligence between graduates of general high schools and those from religious/vocational prayer schools. Additionally, students from sports high schools differed significantly (*p* < 0.013) in rhythmic intelligence, while variations were also found in multiple intelligence domains among graduates of religious/prayer schools, Anatolian/science high schools, vocational schools, and colleges. Logical intelligence showed a marked difference (*p* < 0.044) between graduates of sports high schools and vocational schools, and a significant difference (*p* < 0.013) in naturalistic intelligence was noted between these two groups as well.

The data obtained from the participants were analyzed using One-Way ANOVA and Tukey’s post-hoc tests. As presented in Table 7, the type of sports activity did not yield a statistically significant difference in intelligence domains, except for interpersonal intelligence. Notably, this result appears to favor individuals engaged in individual sports disciplines, who demonstrated relatively higher interpersonal intelligence scores.

**Table 7:** Average of multiple intelligence areas by sport type.

	Sport type	*N*	Mean	SD Deviation	*F*	*p*
Interpersonal	Individual	45	9.25	6.068	3.496	0.034*
	Team	35	6.35	3.352		
	Both	22	8.10	5.427		
	Total	102	8.11	5.338		

*Statistically significant difference *p* < 0.05.

## Discussion

This study revealed statistically significant differences in multiple intelligence domains based on gender, school type, and sport branch among prospective physical education teachers. These findings both align with and diverge from previous research, reflecting the complexity and context-dependency of multiple intelligences. For example, Aytaç (2017) and Vural (2023) found that female participants scored higher in verbal, visual, or kinesthetic intelligence, while Shahzada et al. (2015) and Furnham (2001) reported a male advantage in logical-mathematical intelligence. Similarly, Abacı and Baran (2007) observed a significant difference favoring females in verbal intelligence (*p* = 0.02) but did not report consistent patterns across other domains. Such inconsistencies may stem from variations in sample characteristics, cultural environments, educational systems, or research methodologies.

In terms of gender, our study found that female participants outperformed males in verbal and visual intelligences. Although these differences were statistically significant (*p* < 0.05), the effect sizes (*η*^2^ = 0.06 for verbal; *η*^2^ = 0.04 for visual) indicate small to moderate practical significance. This suggests that gender alone explains only a limited portion of the variance in intelligence profiles, reinforcing the idea that intelligence is not fixed but shaped by experience and context (Kornhaber, 2001; Robinson, 2009).

A noteworthy contribution of this study lies in its focus on prospective physical education teachers a population rarely addressed in MI research, which typically centers on children and adolescents. Interestingly, participants in our study exhibited higher verbal–linguistic intelligence and lower bodily-kinesthetic intelligence, contrary to expectations. This may reflect the increasing theoretical and academic emphasis within higher education programs in physical education, which prioritize classroom-based learning and theoretical coursework over physical development (Altınok, 2008; Kemeç, 2016; Ürgüp and Aslan, 2015). Additionally, the medium-level scores in bodily-kinesthetic intelligence may also reflect the constraints of the COVID-19 pandemic, which restricted physical activity and access to facilities during critical developmental periods (Bayram and Yüceloglu Keskin, 2020; Visser et al., 2006).

Findings regarding school type also provide a nuanced perspective. While Saadatmanesh (2013) reported strong associations between school type and intelligence profiles, our study found limited and context-specific differences. Specifically, graduates of religious high schools displayed slight disadvantages in intrapersonal intelligence, though the effect size was small (η^2^ = 0.03). These results suggest that school type may exert only a modest influence on intelligence development.

Contrary to commonly held assumptions, sport branch was not a significant predictor of bodily-kinesthetic intelligence. Previous literature (Del Pino Medina et al., 2009; Shuadi et al., 2020) suggested that participation in team sports could enhance interpersonal and kinesthetic intelligences. However, our results challenge this view, with a negligible effect size (η^2^ < 0.01). This finding calls into question the assumption that athletic participation inherently develops bodily-kinesthetic intelligence. One potential explanation is that university-level physical education programs may lack sufficient practical or movement-based components. Bayram and Yüceloglu Keskin (2020) argue that university entrance exam criteria for physical education programs may also need to be revisited to better evaluate kinesthetic competencies.

Supporting this view, Zayed et al. (2023) found that although sports practitioners generally scored higher in most intelligence domains, statistically significant differences were limited to bodily-kinesthetic and emotional intelligences. Their findings underscore that while regular engagement in sport can positively impact specific intelligences, this development is not guaranteed across all domains and may depend on the depth and structure of engagement. The cultural and educational context, as shown in their Oman-based study, also plays a vital role in shaping intelligence profiles among sport participants and non-participants.

Furthermore, Tirri and Nokelainen (2008) emphasize that intelligence is shaped by a complex interplay of demographic, socioeconomic, and educational factors an insight our findings strongly support.

Overall, while this study shares some similarities with previous findings (Altınok, 2008; Koçak, 2019), it offers a more contextually grounded and multidimensional understanding of how gender, school type, and sport branch interact with intelligence development in the context of higher education. Gardner’s theory of Multiple Intelligences underscores that intelligence is not a static attribute but a dynamic construct influenced by environment, learning experiences, and individual differences. Our findings confirm this premise and highlight the need for more inclusive and differentiated instructional models in teacher training programs.

From an educational standpoint, these results reinforce the importance of integrating MI theory into teacher education. Effective pedagogy must move beyond a narrow focus on verbal–linguistic and logical-mathematical intelligences to include underrepresented domains such as bodily-kinesthetic, spatial, and interpersonal intelligences (Kornhaber, 2001; Combs et al., 2010). As Robinson (2009) notes, education must cultivate individuality by engaging multiple forms of intelligence. Furthermore, teams composed of individuals with diverse and complementary intelligence profiles tend to function more effectively (West and Borrill, 2023). Accordingly, pre-service teachers should not only identify their dominant intelligences but also learn to translate these strengths into pedagogical strategies (Şuruba-Rusen et al., 2020; Yurt and Polat, 2015).

This reconceptualization of intelligence challenges longstanding assumptions about the dominance of certain intelligence types among physical education students and calls for a more equitable and evidence-based teacher training model. Programs should intentionally foster cognitive diversity by embedding MI-aligned practices, such as role-playing, peer-teaching, reflective assignments, and hybrid instruction, all of which support deeper learning and inclusivity. As Attwood (2022) and Büyükkaragöz et al. (1998) suggest, the success of education depends on the quality of teachers, which in turn depends on the breadth and depth of their cognitive competencies.

### Practical applications

The results of this study reveal that verbal/linguistic intelligence was the most developed domain among physical education teacher candidates, while bodily-kinesthetic intelligence, surprisingly, showed lower scores. This profile underscores the importance of deliberately incorporating kinesthetic and interpersonal learning opportunities within physical education teacher education programs. A substantial body of academic research has explored the relationship between Multiple Intelligences (MI) Theory and teacher education (Shore, 2004; Gul and Rafique, 2017; Al Ardha et al., 2018; Abdelhak and Romaissa, 2022; Xie and Xu, 2022; Gani et al., 2023). The findings of these studies are critical in identifying prospective teachers’ dominant intelligence domains, diversifying instructional methods, and supporting the development of students’ individual strengths and learning needs.

As Saiz-González et al. (2024) emphasized, pre-service teachers’ negative school experiences often stem from rigid teaching styles that overlook diverse learner profiles. Addressing this through more inclusive and multimodal instruction could improve engagement and learning outcomes.

Incorporating hybrid pedagogical models, such as combining student-designed games with student-made materials, as explored by Méndez-Giménez and Garví-Medrano (2025), may enhance bodily-kinesthetic and interpersonal intelligences by fostering active participation, creativity, and social interaction. Moreover, Espinoza-Gutiérrez et al. (2024) highlighted how teaching style significantly influences students’ academic self-concept through the mediation of basic psychological needs, closely linked to interpersonal and intrapersonal intelligences. These findings advocate for teacher education programs that align instructional approaches with students’ motivational and cognitive profiles.

To implement this in training, programs could include practical workshops focused on intelligence-based lesson planning, peer-teaching tasks tailored to multiple intelligence domains, and reflective assignments that help future teachers identify their own dominant intelligences. As Ponce et al. (2025) argue, teacher education should move beyond opinions and incorporate evidence-based strategies, such as the MI framework, to ensure effective, differentiated learning environments. Furthermore, teacher educators must foster inclusive practices, as emphasized by Marcos-Rivero et al. (2024), ensuring that diverse intelligence profiles are not only acknowledged but actively cultivated.

## Conclusion

This study revealed significant variations across multiple intelligence domains, suggesting that these differences may result from personal, sociological, cultural, and psychological factors. Future research in education, pedagogy, and intelligence should adopt more comprehensive and multifaceted approaches to investigate the underlying causes of these variations. Re-examining the hypotheses tested in this study using different research designs, instruments, and larger, more diverse samples could yield more robust and generalizable findings.

Particularly, future investigations should focus on the bodily-kinesthetic intelligence levels of prospective physical education and sports teachers. Research exploring this domain through the implementation of MI-based instructional strategies can offer deeper insights into how such intelligence develops. Including demographic variables such as socioeconomic status and access to extracurricular activities in future studies would further enrich our understanding of intelligence development within teacher education contexts.

Gardner’s theory of Multiple Intelligences (MI) underscores the diversity of cognitive capacities and promotes an individualized and inclusive approach to education. The reliability of MI assessments has improved through empirical tools such as the Multiple Intelligence Profiling Questionnaire (Tirri and Nokelainen, 2008). By examining physical education teacher candidates, a group rarely addressed in MI research this study adds a novel perspective to the existing literature.

One of the most striking findings of this research is that, contrary to expectations, verbal–linguistic intelligence was more developed than bodily-kinesthetic intelligence among the participants. This discrepancy may reflect ongoing structural changes in higher education, particularly the increasing emphasis on academic literacy and theoretical coursework, which may deprioritize kinesthetic development. Additionally, the constraints of the COVID-19 pandemic may have further limited opportunities for physical engagement, contributing to the underdevelopment of bodily-kinesthetic intelligence (Bayram and Yüceloglu Keskin, 2020; Kemeç, 2016; Ürgüp and Aslan, 2015; Altınok, 2008; Visser et al., 2006).

Contrary to earlier studies that reported significant differences in intelligence profiles based on gender and school type, the current research found no statistically significant variations across these variables. This suggests that intelligence development is shaped more by contextual and environmental influences than by static demographic factors. Moreover, the lack of differences across sport branches indicates that mere participation in team or individual sports may not be sufficient to foster specific intelligence domains unless accompanied by intentional pedagogical approaches (Del Pino Medina et al., 2009; Shuadi et al., 2020).

From an educational standpoint, the integration of MI theory into teacher education curricula is imperative. Beyond traditional emphasis on linguistic and logical intelligences, programs should also nurture underrepresented domains such as bodily-kinesthetic, interpersonal, spatial, and musical intelligences (Kornhaber, 2001; Combs et al., 2010). As Robinson (2009) emphasizes, effective education fosters individuality and integrates diverse cognitive strengths. Similarly, teams composed of individuals with complementary intelligence profiles are better positioned to achieve collective goals (West and Borrill, 2023). Therefore, pre-service teachers should be encouraged not only to identify their dominant intelligences but also to translate those strengths into pedagogical practice.

It is critical to expand practical recommendations for teacher training by offering differentiated instructional strategies tailored to specific MI profiles. Examples include:

Verbal–linguistic intelligence: Reflective journaling, structured debates, and analytical discussions.
Logical-mathematical intelligence: Pattern recognition tasks, data analysis exercises, and hypothesis-testing activities.
Bodily-kinesthetic intelligence: Role-playing, task-based movement instruction, and hands-on modeling.
Visual–spatial intelligence: Mind-mapping, tactical diagramming, and video- based performance feedback.
Musical intelligence: Rhythmic drills, musical cues during practice, and performance interpretation.
Interpersonal intelligence: Cooperative learning, peer assessment, and collaborative projects.
Intrapersonal intelligence: Goal-setting reflections, self-assessment portfolios, and mindfulness-based exercises.
Naturalistic Intelligence: Outdoor movement analysis, ecological awareness activities, and nature-based sports contexts.
Existential intelligence: Discussions on ethical dilemmas in sports, reflections on personal purpose and values in teaching, or analyzing philosophical dimensions of competition and fair play.

For example, a lesson may begin with a group discussion on ethical behavior in sports (verbal–linguistic and existential), followed by a collaborative physical task (bodily-kinesthetic and interpersonal), and conclude with a written self-evaluation (intrapersonal). Such designs align with MI theory and promote inclusivity and engagement by addressing both cognitive diversity and deeper reflective capacities.

As Büyükkaragöz et al. (1998) noted, teacher quality is a decisive factor in educational success. The implementation of MI theory in teacher preparation, as Attwood (2022) advocates, should be guided not only by enthusiasm but also by critical and evidence-based perspectives. The present findings resonate with those of Şuruba-Rusen et al. (2020), who examined MI profiles among physical education faculty, and Yurt and Polat’s (2015) meta-analysis, which demonstrated the positive impact of MI based strategies on academic achievement.

To ensure the practical applicability of MI theory, teacher candidates should be actively involved in the development, implementation, and evaluation of MI-based instructional products. This hands-on approach allows future educators to internalize the theory and apply it in diverse classroom settings.

To support a more inclusive and effective teacher training model, MI theory should be integrated into curriculum design. Teacher education programs are encouraged to go beyond traditional verbal and logical emphases by actively fostering bodily-kinesthetic, spatial, interpersonal, and other less-developed intelligences. Instructional strategies could include role-playing, cooperative learning, reflective journaling, and ecological movement analysis, all tailored to specific intelligence domains. Pre-service teachers should be equipped not only to identify their dominant intelligences but also to design learning experiences that align with diverse student needs. Embedding MI based pedagogical models, such as student designed games and hybrid instruction, can enhance both teacher adaptability and learner engagement.

### Limitations

This study has several limitations that should be taken into account when interpreting the findings. First, the research sample was limited to prospective physical education and sports teachers from a specific geographic region, which restricts the generalizability of the results. Future studies involving broader and more diverse populations from different regions and institutional contexts are needed to enhance the inclusiveness of the findings.

Second, the study relied on self-reported data, which may be subject to social desirability bias and inaccuracies in participants’ self-perceptions. Although the Multiple Intelligences Inventory used in the study has been previously validated, self-assessment tools may not fully capture the complexity of individual intelligence domains.

Third, the study did not directly assess the impact of the COVID-19 pandemic on the development of multiple intelligences. While the potential influence of the pandemic on bodily-kinesthetic intelligence was acknowledged, the lack of systematic measurement limits the depth of related interpretations.

Fourth, the descriptive and correlational nature of the research design prevents causal inferences. Therefore, observed relationships between variables should not be interpreted as evidence of direct causation.

Fifth, the study was based exclusively on Gardner’s Theory of Multiple Intelligences. While this theoretical framework is widely respected, incorporating alternative or complementary theories could have provided a more comprehensive perspective.

Lastly, the use of a non-random, convenience sampling method may limit the representativeness of the results. Moreover, the study did not control for potentially confounding variables such as socioeconomic status, extracurricular activity participation, or academic performance, which may have influenced intelligence profiles.

### Recommendations for future research

Considering the limitations discussed in this study, several avenues for future research can be proposed. First, future studies should aim to include larger, more demographically and geographically diverse samples. Randomized selection across multiple institutions will help improve the generalizability of findings and enhance the external validity of research on multiple intelligences. Second, a mixed-methods approach would add depth and richness to future investigations. While self-report instruments provide valuable subjective data, they are susceptible to biases. Incorporating observational methods, instructor and peer evaluations, as well as performance-based assessments, can yield more nuanced insights into the expression and development of different intelligence types, particularly bodily-kinesthetic intelligence. Third, future studies should investigate the long-term consequences of the COVID-19 pandemic on the development of multiple intelligences. Given the widespread restrictions on physical activity during lockdown periods, it is crucial to assess whether and how these constraints have affected the growth of bodily-kinesthetic intelligence, especially among physically active populations like physical education students.

Additionally, causal research designs such as experimental and quasi-experimental methods are necessary to determine whether interventions based on Multiple Intelligences (MI) theory effectively influence learning outcomes. These designs offer stronger internal validity and allow for greater confidence in interpreting cause-effect relationships between educational practices and intelligence development. Moreover, theoretical triangulation should be considered. Integrating MI theory with other educational and cognitive frameworks such as constructivism, social learning theory, or cognitive load theory can provide a more multidimensional interpretation of how intelligences manifest and interact in educational settings.

Lastly, controlling for confounding variables is essential for refining future research. Variables such as socioeconomic status, academic background, and extracurricular involvement can significantly impact intelligence development and should be systematically measured and statistically controlled to isolate the effects of instructional variables.

This study contributes a novel perspective by examining the intelligence profiles of a unique yet underexplored population: prospective physical education teachers. The findings challenge traditional assumptions regarding which intelligence types are dominant in physical education contexts and call attention to the limitations of conventional teacher training approaches that prioritize verbal–linguistic or logical-mathematical abilities.

Teacher education programs should expand their scope to include support for bodily-kinesthetic and other less emphasized intelligences. Such inclusivity will foster the development of well-rounded educators who are capable of meeting the diverse cognitive and developmental needs of today’s learners. Emphasizing a more balanced approach will not only improve teacher effectiveness but also contribute to a more equitable and comprehensive model of human intelligence in education.

In summary, this research underscores the dynamic, contextual, and multifaceted nature of intelligence. It encourages a rethinking of pedagogical models and selection criteria in teacher training programs, advocating for an approach that supports the full range of human cognitive potential.

## Data Availability

The raw data supporting the conclusions of this article will be made available by the authors, without undue reservation.
